# A stigma-reduction intervention targeting abortion and contraceptive use among adolescents in Kisumu County, Kenya: a quasi-experimental study*

**DOI:** 10.1080/26410397.2021.1881208

**Published:** 2023-02-27

**Authors:** Marlene Makenzius, Ulrika Rehnström Loi, Beatrice Otieno, Monica Oguttu

**Affiliations:** a Researcher, Department of Women’s and Children’s Health, and the Department of Global Public Health, Karolinska Institutet, Stockholm, Sweden.; b Researcher, Department of Women’s and Children’s Health, Karolinska Institutet, Stockholm, Sweden; c Project Officer, Kisumu Medical Education Trust (KMET), Kisumu, Kenya; d Executive Director, Kisumu Medical Education Trust (KMET), Kisumu, Kenya

**Keywords:** abortion, adolescents, contraception, Kenya, sexuality education, stigma

## Abstract

This study assessed the effectiveness of a school-based stigma-reduction intervention focusing on stigmatising attitudes towards girls associated with abortion and contraceptive use. In February 2017, two gender-mixed secondary schools (*n* = 1368) in peri-urban areas of Kisumu County, Kenya, were assigned to receive either an 8-hour stigma-reduction intervention over four sessions (intervention school: IS) or standard comprehensive sexuality education (control school: CS). A classroom survey entailing two five-point Likert scales – the 18-item Adolescents Stigmatizing Attitudes, Beliefs and Actions (ASABA) scale, which measures abortion stigma, and the seven-item Contraceptive Use Stigma (CUS) scale – was conducted to collect data at baseline, 1-month and 12-months after the intervention. The intervention was to be considered effective if a mean score reduction of 25% was achieved for both the ASABA (primary outcome) and the CUS (secondary outcome) at the IS between baseline and 12-month follow-up. 1207 (IS = 574; CS = 633) students were included in analyses at 1-month follow-up, and 693 (IS = 323; CS = 370) at 12-months (the final-year students had left school). A decrease in mean score on both scales was observed at 1-month at both schools. At 12-months, the score decrease was 30.1% at the IS and 9.0% at the CS for ASABA, and 27.3% at the IS and 7.9% at the CS for CUS. At the IS, the score decrease for ASABA between baseline and 12-months was 23.3% among girls and 31.2% among boys; for CUS, the decrease was 27.3% and 24.3%, respectively. ASABA and CUS were positively correlated (*r* = 0.543; *p* < 0.001), implying a broader perspective on reproductive stigma. A four-session, school-based stigma-reduction intervention could lead to transformed values and attitudes towards gender norms among adolescents regarding abortion and contraceptive use. Stigma associated with abortion and contraception should become a priority for high-quality CSE programmes.

## Résumé

Cette étude a évalué l’efficacité d’une intervention de réduction de la stigmatisation en milieu scolaire, en se centrant sur les attitudes stigmatisantes à l’égard de filles associées à l’avortement et l’emploi de contraception. En février 2017, deux écoles secondaires mixtes (*n* = 1368) situées dans des zones périurbaines du comté de Kisumu, Kenya, ont été choisies pour faire l’objet soit d’une intervention de réduction de la stigmatisation de huit heures sur quatre séances (école de l’intervention = EI) soit d’une éducation complète à la sexualité standard (école témoin = ET). Une enquête a été réalisée dans les classes au moyen de deux échelles de Likert avec cinq options de réponse – l’échelle sur les attitudes, les croyances et les actions stigmatisantes des adolescents en 18 items (ASABA), qui mesure la stigmatisation liée à l’avortement, et l’échelle en sept items sur la stigmatisation due à l’emploi de contraceptifs (CUS) – afin de recueillir des données pendant la période de référence, un mois et 12 mois après l’intervention. L’intervention devait être considérée comme réussie si une réduction du score moyen de 25% était obtenue aussi bien pour l’échelle ASABA (résultat primaire) que l’échelle CUS (résultat secondaire) dans l’école de l’intervention entre la période de référence et le suivi à 12 mois. 1207 élèves (EI = 574; ET = 633) ont été inclus dans les analyses pour le suivi à un mois, et 693 (EI = 323; ET = 370) pour le suivi à 12 mois (les élèves de dernière année avaient quitté l’école). Une diminution du score moyen des deux échelles a été observée un mois après dans les deux écoles. Douze mois après, la diminution du score était de 30.1% dans l’EI et de 9.0% dans l’ET pour l’échelle ASABA, et de 27.3% à l’EI et de 7.9% à l’ET pour l’échelle CUS. Dans l’EI, la diminution du score pour ASABA entre la période de référence et le suivi à 12 mois était de 23.3% chez les filles et de 31.2% chez les garçons; pour l’échelle CUS, la diminution était de 27.3% et 24.3% respectivement Une corrélation positive a été observée entre les échelles ASABA et CUS (*r* = 0.543, *p* < 0.001), laissant supposer une perspective élargie sur la stigmatisation liée à l’emploi de contraceptifs. Une intervention de réduction de la stigmatisation en milieu scolaire sur quatre séances pourrait aboutir à une transformation des valeurs et des attitudes face aux normes de genre parmi les adolescents concernant l’avortement et l’emploi de contraceptifs. La stigmatisation associée à l’avortement et la contraception devrait devenir une priorité des programmes de qualité d’éducation complète à la sexualité.

## Resumen

Este estudio evaluó la eficacia de una intervención escolar de reducción del estigma, enfocada en actitudes estigmatizantes hacia niñas asociadas con el aborto y el uso de anticonceptivos. En febrero de 2017, se asignaron dos escuelas secundarias con estudiantes de ambos géneros (*n* = 1368) en zonas periurbanas del Condado de Kisumu, en Kenia, para que recibieran una intervención de reducción del estigma, de 8 horas de duración, en cuatro sesiones (escuela de intervención: EI) o educación integral en sexualidad (EIS) estándar (escuela de control: EC). Se realizó una encuesta en el aula, que implicó dos escalas Likert de cinco puntos –una escala de 18 ítems sobre actitudes, creencias y acciones estigmatizantes de adolescentes (ASABA, por sus siglas en inglés), que mide el estigma del aborto, y una escala de siete ítems sobre el estigma del uso de anticonceptivos (CUS)– para recolectar datos de base, 1 mes y 12 meses después de la intervención. La intervención se consideraría eficaz al alcanzar un puntaje medio de 25% para ambas escalas, ASABA (resultado principal) y CUS (resultado secundario) en la EI entre la línea de base y el seguimiento a los 12 meses. Se incluyó a 1207 (EI = 574; EC = 633) estudiantes en los análisis del seguimiento al mes, y 693 (EI = 323; EC = 370) a los 12 meses (año final cuando los estudiantes habían terminado la escuela). En ambas escuelas se observó una disminución del puntaje medio de ambas escalas 1 mes después de la intervención. A los 12 meses, la disminución del puntaje fue de 30.1% en la EI y de 9.0% en la EC para ASABA, y de 27.3% en la EI y 7.9% en la EC para CUS. En la EI, la disminución del puntaje para ASABA entre la línea de base y a los 12 meses fue de 23.3% entre las niñas y 31.2% entre los niños; para CUS, la disminución fue de 27.3% y 24.3%, respectivamente. ASABA y CUS estaban correlacionadas positivamente (*r* = 0.543; *p* < 0.001), lo cual implica una perspectiva más amplia del estigma reproductivo. Una intervención escolar de reducción del estigma, realizada en cuatro sesiones, podría propiciar una transformación de valores y actitudes hacia las normas de género entre adolescentes respecto al aborto y el uso de anticonceptivos. El estigma asociado con el aborto y la anticoncepción deben ser una prioridad para los programas de EIS de alta calidad.

## Introduction

Unintended pregnancy (unwanted or mistimed) is a preventable public health concern, particularly among adolescents and young people in low- and middle-income countries (LMICs). At least 10 million unintended pregnancies occur annually in LMICs among this group.^1^ Unintended pregnancies contribute to 73.3 million abortions globally every year among women and adolescent girls aged 15–49 years.^2^

In LMICs, morbidity and mortality due to unsafe abortions are substantial.^3^ Additional adverse outcomes of unsafe abortion are social consequences and economic consequences, including the direct cost of providing post-abortion care and the long-term cost of lasting health problems and lost productivity for families, communities and the country.^4^ The provision of high-quality, age-appropriate comprehensive sexuality education (CSE) from an early age, together with contraceptive-promoting interventions, has the potential to reduce unintended pregnancies among adolescent girls.^5^ In 2018, UNESCO, together with UNAIDS, UNFPA, UNICEF, UN Women and WHO, published the revised International Technical Guidance on Sexuality Education with key concepts, topics and learning objectives. The revised guidance provides a strong focus on gender and human rights, and includes a chapter on stigma towards women and men living with HIV/AIDS. However, abortion stigma and contraceptive use stigma are left out.^6^

In Kenya, 23% of the total population comprises adolescents aged 10–19 years.^7^ Contraceptive use is low, and only 37% of sexually active female adolescents use modern contraceptive methods. The unmet need for contraception among married adolescent girls was 23% in 2014, which is higher than the national average of 18% among women of reproductive age (15–49 years). The 2014 Kenya Demographic and Health Survey showed that one in five adolescent girls aged 15–19 years is either pregnant with their first child or already a mother.^7^ The adolescent pregnancy rate in Kenya stands at 18%, with an adolescent fertility rate of 96 per 1000 women aged 15–19 compared with the regional (sub-Saharan Africa) adolescent fertility rate of 101 per 1000 women aged 15–19.^8^ In Kenya, most adolescent pregnancies are unintended and are more likely to occur among girls of low economic status and with low levels of education.^9^

Unintended pregnancy is one of the major reasons for induced abortion among young women in Kenya,^10^ where the national estimate of abortions is high: 48 abortions per 1000 women of reproductive age,^11^ compared with a regional estimate of 31 abortions per 1000 women of reproductive age in sub-Saharan Africa.^12^ Most abortions in Kenya are considered unsafe, due to restrictive abortion legislation and limited access to healthcare services.^13^ Despite the fact that safe and effective interventions exist for the termination of pregnancy, unsafe abortions are still a major contributor to maternal morbidity and mortality in Kenya. In 2014, unsafe abortions accounted for 35% of maternal deaths, compared with a global average of 13%.^7^

Early and unintended pregnancies can be prevented by providing age-appropriate, high-quality CSE that promotes gender equality, equitable social norms, delayed sexual debut and access to healthcare services to ensure the provision of modern contraceptives.^14^ CSE can provide a structured opportunity for students to gain evidence-based knowledge and practical skills, to explore their values and attitudes, to promote gender equality, dignity and respect for others, and to make informed choices about their sexual lives.^14,15^

In 2013, the Kenyan government signed a ministerial commitment to scale-up rights-based CSE^16^ with the aim of reducing adolescent pregnancies and abortions among young women nationally.^9,17^ Despite the Kenyan government’s support for school-based CSE, contraceptive use among adolescents is low, and adolescent pregnancy rates are still high.^7^ Access to high-quality information and education on sexual and reproductive health and rights for young people in Kenya is still difficult, as the national CSE policy is limited in scope. The CSE curriculum is purely academic, focusing on biology, and is considered weak because information on contraceptives, reproduction, abortion and sexual health services is ignored.^18^ Furthermore, stigmatising values and attitudes are communicated to students, with fear-inducing and judgemental messages emphasising that sex is dangerous and immoral for young people; thus, there is a need for improved CSE in secondary schools.^18^ Teachers in Kenya are not trained to provide CSE to all students and they face numerous challenges in the classroom. Lack of knowledge of abortion services and contraceptive methods, perceived community opposition, lack of materials and resources, and their own discomfort all affect the quality of CSE delivered.^18^ Implementation of the national CSE policy in Kenya has been demanding, as the country lacks official legislation to enforce it.^18^

While there is evidence that adolescents face stigmatising attitudes from teachers and peers, which may be a major barrier to their use of modern contraceptive methods,^19,20^ interventions to reduce the stigma around abortion and contraceptive use by adolescents in LMICs have not been studied extensively.^21,22^ The aim of the present study was to assess whether a school-based stigma-reduction intervention focusing on stigmatising attitudes towards girls associated with abortion and/or contraceptive use, that promotes gender equality, equitable norms and value clarification, could be effective in transforming attitudes and beliefs regarding abortion and contraceptive use among secondary-school students in Kisumu County, Kenya.

## Methods

### Study design and participants

This was a 12-month, school-based, quasi-experimental study with a controlled before–after design (ClinicalTrial.gov: NCT03065842), reported in accordance with the Transparent Reporting of Evaluations with Nonrandomized Designs (TREND) guidelines.^23^ Data collection for the two-arm study was conducted at two mixed-gender secondary day-schools in Kisumu County, located in the western region of Kenya, from 1 February 2017 to 31 March 2018. Kisumu County is one of 47 counties nationally and has a population of 1,156,000 people.^24^ It is a low-resource setting in which 39% of the total population are below the age of 15 years, and 12% are aged 15–19.^25^

A detailed description of the students and study site are presented elsewhere.^26^ Based on a regional sampling frame of 21 schools, all of which conducted Christian religious education, five schools were considered to meet the inclusion criteria to facilitate comparability of communities with respect to baseline data. The inclusion criteria were as follows: public, secondary day-schools of mixed gender; in suburban areas within Kisumu County; hosting a minimum of 300–400 students; and conducting Christian religious education (as this holds a key position in the country and within the Kenyan school curricula). Of the five schools that met the inclusion criteria, one school declined to participate. In the presence of the research team, the study coordinator (MO) at Kisumu Medical and Education Trust (KMET) wrote the names of the four remaining schools on four pieces of paper, which were then folded and mixed, and the principal statistician manually drew one piece of paper at a time. It was pre-specified that the school on the first piece of paper to be drawn would be assigned to be the intervention school, and the school on the second piece of paper would be the control school; the other two pieces of paper were read for transparency. The selected schools hosted, in total, 1368 students (aged 13–21 years), divided into four grades. Under the current system, students attend secondary school for four years. In Kenya, the age of students cannot be related to a specific grade because socioeconomic factors, health factors and social norms often interfere with school start, and lead to occasional and permanent dropouts. The distance between the two schools was approximately 5 km. Students from both schools came from low-income communities and were, therefore, considered to be in a low socioeconomic position.

### Stigma-reduction intervention

The stigma-reduction programme lasted for three weeks (8–9 h, divided into four sessions) and was provided to mixed-gender classes according to the school system. The basic content was the same for all students; however, the discussions were driven by the participants and, therefore, may have differed between the classes due to the students’ age, maturity and sexual experience. The intervention involved a combination of strategies recommended to transform students’ attitudes, values and predicted behaviours, including on abortion and contraceptive use. The intervention followed the International Planned Parenthood Federation (IPPF) guide *How to Educate about Abortion*, which has a focus on unintended pregnancy, abortion and contraception.^27^ The educators used interactive learning models with opportunities to discuss misconceptions, attitudes and beliefs through group discussions, chart-writing, role-playing, lecturing, presentations and anonymous question-and-answer sessions. The goal of the value clarification was to help students identify their own and others’ misconceptions and myths, and the realities surrounding abortion and contraception. No incentives were provided to the school teachers or the students.

### Participant and public involvement

The intervention was tailored through formative research with teachers, school principals, students, health workers from youth-friendly clinics (public, private and faith-based facilities), healthcare providers from public hospitals, representatives from the Ministry of Education and the Ministry of Health, religious leaders, and local and national NGOs, as well as local and international researchers. Several strategies were used to inform these groups of the topic, such as written information distributed through different channels (which also reached parents), workshops and focus group discussions with stakeholders and authorities.^28^

### Delivery of intervention

A team of 42 educators was used: six staff from KMET, 12 volunteers, four teachers, and 20 peer-educators at the intervention school were trained (January–February 2017) to provide the intervention. Five training sessions were conducted: two at the intervention school and three at KMET.

The control school received the standard CSE programme, as outlined in the curriculum and by CSE policy in Kenya. The research team had no control over what was provided or not provided to the students at the control school. However, it is documented that CSE in Kenya is disconnected from the needs of young people and is influenced by a moralistic approach; furthermore, teachers lack training and support in providing CSE.^21^

Trained research assistants directed data collection at both schools at each timepoint: baseline; 1 month after the intervention; and 12 months after the intervention (endpoint). A brief introduction was provided regarding the questionnaires and the consent forms, and the students completed them in classrooms during a period of 15–40 min and then returned the questionnaires, sealed in an envelope, to an assistant.

### Outcomes

Outcomes were assessed at baseline, 1-month follow-up and 12-month follow-up using two validated scales. In the pre-work for the present study, the original Stigmatizing Attitudes, Beliefs and Actions (SABA) scale ^29^ was modified and validated as the 18-item Adolescents Stigmatizing Attitudes, Beliefs and Actions (ASABA) scale to fit the adolescent population within the current study.^30^ Similarly, the seven-item Contraceptive Use Stigma (CUS) scale was developed and validated within the pre-work of the project.^30^ Responses to the two surveys were specified on a five-point Likert scale ranging from strongly disagree (1) to strongly agree (5). The main outcomes were score changes on the ASABA scale and the CUS scale between baseline and 12-month follow-up. An additional secondary outcome was contraceptive use, which will be presented elsewhere with additional qualitative data. The intervention was to be considered effective if there was a mean score reduction of 25% on both the ASABA (primary outcome) and the CUS (secondary outcome) at the intervention school between baseline and 12-month follow-up. The score decrease was expected to be greater at the intervention school than at the control school. This was a new research area and, therefore, we had to estimate what could be a reasonable reduction. Representatives such as teachers responsible for CSE and students at secondary schools participated together with the research team in the estimation process, as no literature could be identified on the topic.

### Sample size

Because the sampling units were schools, the sample size was arbitrarily determined based on the size of the schools. All female and male students registered on 31 January 2017 at the two schools selected from the sampling frame were eligible to participate and were allocated to study arms at baseline (*n* = 1368). Attrition was mainly due to illness or unpaid school fees, and it was known that the final-year students would not be available for the 12-month follow-up.^25^

### Statistical analyses

Descriptive statistics of the study population were calculated by estimating means, standard deviations (SD) and proportions. The Pearson chi-squared test for categorical variables and the independent *t*-test (two-tailed) for continuous variables were used to compare baseline characteristics between participants at the intervention school and those at the control school. Data collected were analysed using either *t*-test (two-tailed) or repeated-measures analysis of variance (ANOVA) within and between subjects at baseline, 1-month and 12-month follow-up. The subscales and full scales were analysed using interclass correlation and Cronbach alpha coefficients to evaluate the correlation between scales, and internal consistency and reliability, respectively. The paired-sample *t*-test (two-tailed) was conducted to compare mean score change between baseline and 12-month follow-up at the intervention school. The same analysis was also conducted at the control school. The percentage score reduction was calculated (using raw data) by subtracting the 12-month value from the baseline value, dividing by the baseline value and multiplying by 100. The paired-sample *t*-test (two-tailed) was also used for the comparison of stigma score at the intervention school between baseline and 12-month follow-up within gender and within age groups, and the independent-sample *t*-test (two-tailed) was used for comparisons between genders and between age groups. All statistical analyses were performed using SPSS, version 27.0.

### Ethical considerations

The study was approved by the Jaramogi Oginga Odinga Teaching and Referral Hospital Ethical Review Committee (ERC.1B/VOL.I/263; 31 May 2016) and the Ministry of Education, State Department of Basic Education (CDE/KSM/GA/20/13/115; 18 January 2017). A research licence was obtained from the National Commission for Science, Technology and Innovation, Nairobi, Kenya (NACOSTI/P/18/68231/25970). All participants aged 18 years and older provided verbal and written informed consent for inclusion in the study. Participants under the age of 18 were initially required to have written parental consent to participate; however, this was a challenging procedure to perform, and therefore a signed consent from the class teacher, in addition to pupil assent, was accepted. The protocol change was verbally approved at the time by the Jaramogi Oginga Odinga Teaching and Referral Hospital Ethical Review Committee, and later formalised in a written letter (IERC/JOORTH/412/2021; 6 May 2021).

## Results

**Figure 1.**
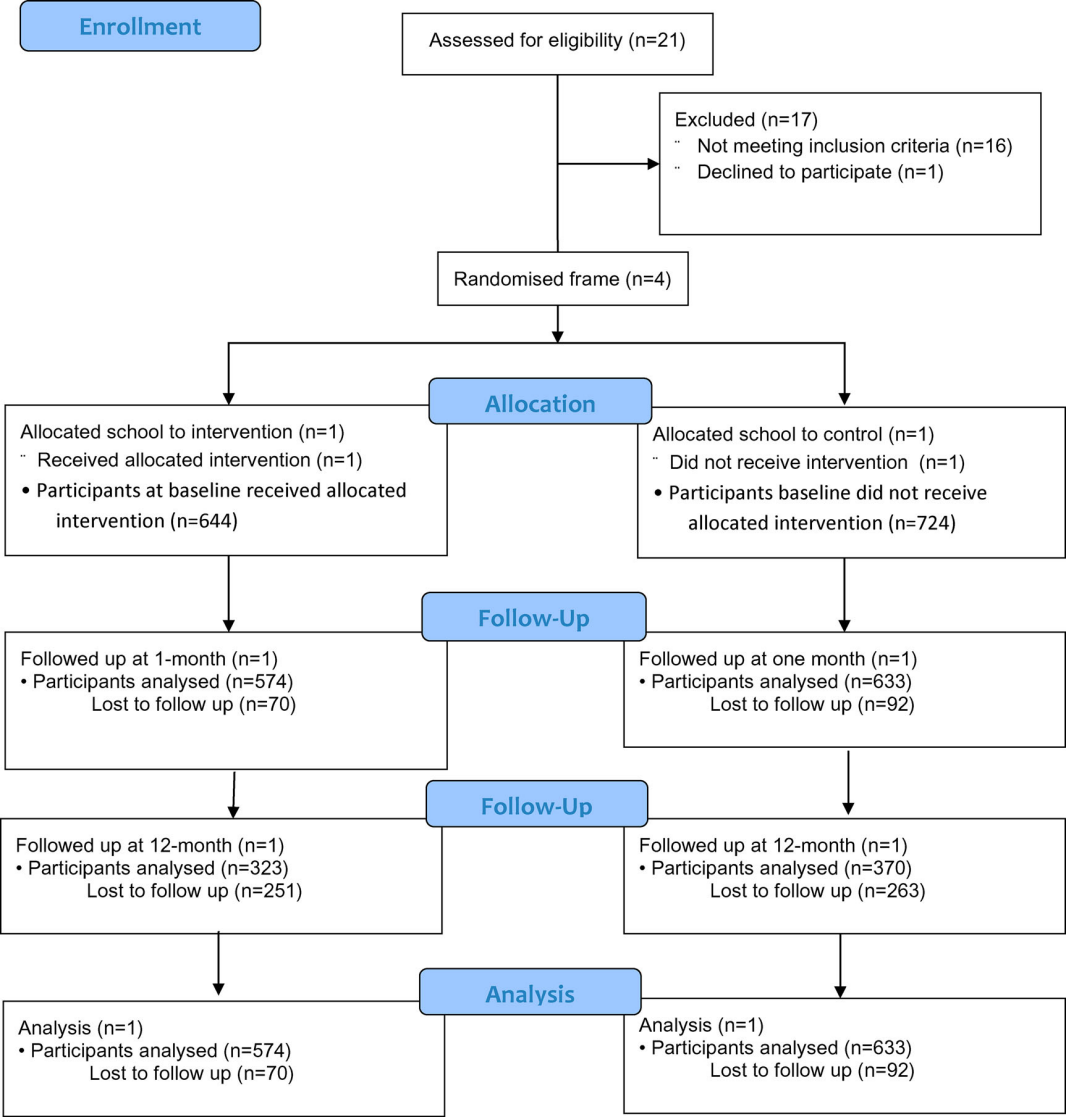
Flow diagram.

As shown in Figure 1, baseline data were collected for 1368 students (644 at the intervention school and 724 at the control school). Data from 1207 (88.2%) students were analysed at one-month follow-up; data from 693 (50.6%) students were analysed at 12-month follow-up: 323 at the intervention school and 370 at the control school.

Table 1 shows the characteristics of students at the intervention school and the control school. At baseline, mean (SD) ages were 16.4 (1.5) years at the intervention school and 16.9 (1.5) years at the control school. There was evidence of differences at baseline between the two schools in mean scores for all three subscales and the full ASABA scale, but not for CUS scores (Table 1).

**Table 1.** Comparison of student characteristics between the intervention school and the control school^a^

	Total	IS	CS	*p*-value
**Baseline**	**(*N* = 1207)**	**(*n* = 574)**	**(*n* = 633)**	** **
Female, *n* (%)	618 (51·5%)	293 (51·4%)	325 (51·6%)	0·949
Male, *n* (%)	582 (48·5%)	277 (48·6%)	305 (48·4%)	
Mean (SD) age, y	** **	16·4 (1·5)	16·9 (1·5)	<0.001
**Mean (SD) subscales of ASABA**				
Negative stereotypes (8 items)	** **	3·41 (0·76)	3·50 (0·72)	0·037
Discrimination and exclusion (7 items)	** **	1·79 (0·65)	1·90 (0·67)	0·005
Potential contagion (3 items)	** **	1·83 (0·78)	1·95 (0·73)	0·005
**Mean (SD) full scale**				
Total 18-item ASABA	** **	2·52 (0·55)	2·62 (0·52)	<0·001
Total 7-item CUS	** **	2·68 (0·83)	2·75 (0·74)	0·094
**1-month follow-up**	**(*N* = 1207)**	**(*n* = 574)**	**(*n* = 633)**	** **
Female *n* (%)	618 (51·5%)	293 (51·4%)	325 (51·4%)	0·949
Male *n* (%)	582 (48·5%)	277 (48·6%)	305 (48·4%)	
**12-month follow-up**	**(*N* = 693)**	**(*n* = 323)**	**(*n* = 370)**	** **
Female *n* (%)	363 (52·4%)	170 (52·6%)	193 (52·2%)	0·902
Male *n* (%)	330 (47·6%)	153 (47·4%)	177 (47·8%)	

^a^ Baseline sample size included only participants who were available at 1-month follow-up.

Table 2 shows the distribution of mean scores for each item in the ASABA scale at different timepoints. At the intervention school, the mean score decreased for each ASABA item between baseline and 1-month follow-up, and there was a large decrease in mean score for each item between baseline and 12-month follow-up (range, 13.5–40.1%). At the control school, the mean score decreased for most ASABA items between baseline and 1-month follow-up, and there was a decrease for all items between baseline and 12-month follow-up (range, 3.5–16.0%). There was a decrease in mean score for each item of the CUS scale between baseline and 1-month follow-up at the intervention school (Table 3), and a large decrease between baseline and 12-month follow-up (range, 17.7–31.1%). At the control school, the mean score for most CUS items decreased between baseline and 1-month-follow-up, and for all items between baseline and 12-month follow-up (range, 3.1–10.4%).

**Table 2.** Distribution of mean scores for each question of the ASABA scale at the intervention school and the control school

ASABA scale item (Likert 1–5)	Intervention school						Control school					
	Baseline (*n* = 574)	1 month (*n* = 574)	12 months (*n* = 323)	F-value	*p*-value	Score decrease^a^ %	Baseline (*n* = 633)	1 month (*n* = 633)	12 months (*n* = 370)	F-value	*p*-value	Score decrease^a^ %
	Mean (SD)						Mean (SD)					
A girl who has an induced abortion is committing a sin.	4·46 (1·09)	2·73 (1·51)	2·67 (1·50)	289·244	<0·001	40·1	4·50 (1·06)	4·26 (1·28)	4·28 (1·20)	7·199	<0·001	4·9
Once a girl has had one abortion, she will make it a habit.	3·31 (1·34)	2·30 (1·23)	2·11(1·13)	130·907	<0·001	36·3	3·38 (1·31)	3·22 (1·30)	3·06 (1·32)	7·048	<0·001	9·5
A girl who has had an abortion cannot be trusted.	2·74 (1·38)	2·06 (1·13)	1·89 (1·08)	65·245	<0·001	31·1	3·05 (1·42)	2·76 (1·32)	2·60 (1·33)	14·421	<0·001	14·8
A girl who has had an abortion brings shame to her family.	3·85 (1·32)	3·06 (1·44)	2·55 (1·37)	101·330	<0·001	33·8	3·92 (1·23)	3·65 (1·35)	3·61 (1·29)	9·317	<0·001	7·9
The health of a girl who has had an abortion is never as good as it was before the abortion.	3·80 (1·33)	2·89 (1·45)	2·56 (1·45)	99·375	<0·001	32·6	3·78 (1·36)	3·56 (1·37)	3·62 (1·31)	4·212	0·015	4·2
A girl who has had an abortion might be a bad influence on other girls.	3·26 (1·44)	2·60 (1·37)	2·19 (1·22)	68·911	<0·001	32·8	3·29 (1·42)	3·18 (1·38)	3·17 (1·33)	1·390	0·249	3·6
A girl who has had an abortion will be a bad mother.	2·37 (1·27)	1·78 (1·04)	1·51 (0·85)	73·539	<0·001	36·3	2·62 (1·36)	2·52 (1·25)	2·20 (1·15)	12·707	<0·001	16
A girl who has had an abortion brings shame to her community.	3·51 (1·41)	2·80 (1·43)	2·21 (1·28)	94·121	<0·001	37	3·46 (1·36)	3·38 (1·38)	3·19 (1·33)	4·559	0·011	7·8
A girl who has had an abortion should be prohibited from going to religious services.	1·48 (0·85)	1·35 (0·72)	1·28 (0·61)	7·462	<0·001	13·5	1·54 (0·88)	1·66 (1·04)	1·42 (0·79)	7·234	<0·001	7·8
A girl who has had an abortion should be teased so that she will be ashamed about her decision.	2·02 (1·18)	1·65 (0·96)	1·56 (0·79)	28·034	<0·001	22·8	2·14 (1·29)	2·27 (1·26)	1·97 (1·17)	6·400	<0·001	7·9
A girl should be disgraced in my community if she has had an abortion.	1·88 (1·03)	1·64 (0·94)	1·51 (0·73)	17·686	<0·001	19·7	2·09 (1·17)	2·11 (1·13)	1·88 (0·97)	5·821	<0·001	10
A man should not marry a woman who has had an abortion.	2·04 (1·19)	1·72 (1·03)	1·54 (0·80)	25·525	<0·001	24·5	2·06 (1·16)	2·17 (1·20)	1·92 (0·98)	5·129	<0·001	6·8
A girl who has had an abortion should no longer be associated with	1·78 (1·03)	1·58 (0·92)	1·50 (0·77)	11·394	<0·001	15·7	1·90 (1·12)	2·00 (1·15)	1·87 (1·02)	2·070	0·127	1·6
A girl who has had an abortion should be pointed fingers at so that other people would know what she has done	1·67 (1·01)	1·59 (1·03)	1·51 (0·80)	3·106	0·045	9·6	1·81 (1·09)	2·00 (1·22)	1·73 (1·05)	6·931	<0·001	4·4
A girl who has had an abortion should not be treated the same as everyone else.	1·68 (1·11)	1·56 (0·94)	1·50 (0·83)	3·640	0·026	10·7	1·75 (1·04)	1·94 (1·14)	1·61 (0·85)	11·480	<0·001	8
A girl who has had an abortion can make other people fall ill or get sick.	1·59 (0·99)	1·45 (0·81)	1·37 (0·76)	7·378	<0·001	13·8	1·69 (1·04)	1·68 (1·02)	1·53 (0·93)	3·393	0·034	9·5
A girl who has had an abortion should be isolated from other people in the community for at least 4 weeks after having the abortion.	1·96 (1·11)	1·64 (0·94)	1·53 (0·77)	24·842	<0·001	21·9	2·20 (1·13)	2·06 (1·08)	2·06 (1·05)	3·077	0·046	6·4
If a boy has sex with a girl who has had an abortion, he will most likely become infected with a disease.	1·94 (1·16)	1·73 (1·07)	1·56 (0·84)	13·862	<0·001	19·6	1·96 (1·11)	2·03 (1·19)	1·90 (1·08)	1·561	0·210	3·1

^a^ Score decrease (baseline value minus 12-month value, divided by baseline value and multiplied by 100) calculated using raw data.

**Table 3.** Distribution of mean scores for each question of the CUS scale at the intervention school and the control school

CUS scale item (Likert 1-5)	Intervention school						Control school					
	Baseline (*n* = 574)	1 month (*n* = 574)	12 months (*n* = 323)	F-value	*p*-value	Score decrease, % ^a^	Baseline (*n* = 633)	1 month (*n* = 633)	12 months (*n* = 370)	F-value	*p*-value	Score decrease, %^a^
	Mean (SD)						Mean (SD)					
A girl who uses a contraceptive method is promiscuous	3·05 (1·47)	2·36 (1·32)	2·10 (1·26)	59·927	<0·001	31·1	3·11 (1·38)	3·00 (1·34)	2·94 (1·30)	1·968	0·140	8·1
A girl who uses a contraceptive method will encourage others to be promiscuous	3·09 (1·41)	2·45 (1·27)	2·19 (1·19)	57·530	<0·001	29·1	3·20 (1·39)	3·10 (1·32)	3·10 (1·31)	1·129	0·324	3·1
A girl cannot decide for herself if to use a contraceptive method.	2·36 (1·25)	2·08 (1·21)	1·77 (0·99)	26·737	<0·001	25	2·37 (1·27)	2·51 (1·30)	2·16 (1·18)	8·027	<0·001	8·9
A married woman is more deserving of a contraceptive method than an unmarried woman.	2·49 (1·34)	2·21 (1·29)	1·89 (1·09)	22·858	<0·001	24·1	2·68 (1·38)	2·64 (1·35)	2·47 (1·32)	2·894	0·056	7·8
A girl who uses contraceptives will have a problem when she decides to get pregnant.	3·22 (1·44)	2·47 (1·36)	2·24 (1·24)	65·972	<0·001	30·4	3·16 (1·46)	3·07 (1·37)	3·05 (1·37)	0·814	0·443	3·5
A girl who carries condoms is likely to have many sexual partners.	2·83 (1·50)	2·35 (1·40)	2·03 (1·26)	36·238	<0·001	28·3	2·89 (1·48)	2·84 (1·45)	2·59 (1·45)	5·052	0·007	10·4
A girl should not insist on using a condom, it is for the man to decide whether to use a condom or not.	1·74 (1·11)	1·64 (1·05)	1·43 (0·83)	8·896	<0·001	17·7	1·86 (1·23)	2·10 (1·28)	1·72 (1·18)	10·818	<0·001	7·5

^a^ Score decrease (baseline value minus 12-month value, divided by baseline value and multiplied by 100) calculated using raw data.

Table 4 shows positive interclass correlations between the three subscales of the ASABA, the full ASABA scale and the CUS scale. The correlation lies between 0.322 and 0.866. Thus, the ASABA scale is positively related to the CUS scale (*r* = 0.543; *p* < 0.001).

**Table 4.** Interclass correlations for the three subscales and the full ASABA scale

	Negative stereotypes	Discrimination and exclusion	Potential contagion	Total ASABA Score	CUS scale
**Subscales of ASABA**					
Negative stereotypes	1	0·387*	0·322*	0·866*	0·463*
Discrimination and exclusion		1	0·519*	0·767*	0·410*
Potential contagion			1	0·622*	0·378*
**Full scales**					
ASABA				1	0·543*
CUS					1

*p < 0·01.

Table 5 presents the reliability analysis for the subscales of the ASABA, the full ASABA scale and the CUS scale. Cronbach alpha ranges from 0.451 to 0.761, indicating acceptable consistency – with the exception of “potential contagion” (0.451), which contained only three items.

**Table 5.** Internal consistency coefficients for subscales of the ASABA scale, the full ASABA scale and the CUS scale

	No. of items	Cronbach alpha
**Subscales of ASABA**		
Negative stereotypes	8	0·686
Discrimination and exclusion	7	0·709
Potential contagion	3	0·451
**Full scales**		
ASABA	18	0·761
CUS	7	0·656

There were decreases in mean score for the three ASABA subscales at both schools (Table 6). For the “negative stereotypes” subscale, there was a decrease of 36.9% at the intervention school and 8.5% at the control school between baseline and 12-month follow-up; for the “discrimination and exclusion” subscale, there was a decrease of 19.6% at the intervention school and 8.8% at the control school; and for the “potential contagion” subscale, there was a decrease of 21.8% at the intervention school and 9.0% at the control school. In terms of the primary outcome, there was a 30.1% decrease in mean score for the full-scale ASABA at the intervention school between baseline and 12-month follow-up, and a 9.0% decrease at the control school. With regard to the secondary outcome, there was a 27.3% decrease in mean CUS score at the intervention school between baseline and 12-month follow-up, and a 7.9% decrease at the control school.

**Table 6.** Mean (SD) scores and score changes for intervention school and control school^a^

Subscales of ASABA	Baseline (1) (*n* = 574)	1 month (2) (*n* = 574)	12 months (3) (*n* = 323)	Mean difference (95% CI) (1–2)	Mean difference (95% CI) (1–3)	Mean difference (95% CI) (2–3)	Change (95% CI) ^b^
**Intervention school**							
Negative stereotypes	3·54 (0·71)	2·97 (0·86)	2·75 (0·94)	0·57 (0·49, 0·66) (*p* < 0·001)	0·78 (0·69,0·88) (*p* < 0·001)	0·21 (0·13, 0·29) (*p* < 0·001)	−1·29 (−1·40, −1·19) (*p* < 0·001)
Discrimination and exclusion	1·90 (0·67)	1·79 (0·70)	1·65 (0·55)	0·10 (0·03, 0·17) (*p* = 0·002)	0·25 (0·18, 0·32) (*p* < 0·001)	0·15 (0·08, 0·22) (*p* < 0·001)	−0·36 (−0·43, −0·28) (*p* < 0·001)
Potential contagion	1·95 (0·77)	1·77 (0·75)	1·67 (0·66)	0·18 (0·10, 0·26) (*p* < 0·001)	0·28 (0·20, 0·37) (*p* < 0·001)	0·10 (0·03, 0·19) (*p* = 0·005)	−0·40 (−0·50, −0·30) (*p* < 0·001)
**Full scales**							
ASABA	2·64 (0·53)	2·31 (0·64)	2·14 (0·61)	0·33 (0·27, 0·38) (*p* < 0·001)	0·50 (0·43, 0·56) (*p* < 0·001)	0·17 (0·12, 0·23) (*p* < 0·001)	−0·78 (−0·85, −0·71) (*p* < 0·001)
CUS	2·76 (0·75)	2·47 (0·80)	2·14 (0·61)	0·30 (0·21, 0·38) (*p* < 0·001)	0·63 (0·54, 0·71) (*p* < 0·001)	0·33 (0·26, 0·41) (*p* < 0·001)	−0·73 (−0·83, −0·63) (*p* < 0·001)
**Control school**							
Subscales of ASABA	Baseline (1) (*n* = 633)	1 month (2) (*n* = 633)	12 months (3) (*n* = 370)	Mean difference (1−2) CI: (LB−UB)	Mean difference (1−3) CI: (LB−UB)	Mean difference (2−3) CI: (LB−UB)	**Change (95% CI) ^b^**
Negative stereotypes	3·55 (0·70)	3·35 (0·73)	3·25 (0·76)	0·20 (*p* < 0·001) (0·10, 0·30)	0·30 (*p* < 0·001) (0·20, 0·41)	0·10 (*p* = 0·065) (−0·00, 0·21)	−0·30 (−0·38, −0·22) (*p* < 0·001)
Discrimination and exclusion	1·94 (0·69)	1·98 (0·73)	1·79 (0·60)	−0·03 (−0·13, 0·06) (*p* > 0.99)	0·15 (0·05, 0·25) (*p* = 0·001)	0·18 (0·08, 0·29) (*p* < 0·001)	−0·16 (−0·24, −0·09) (*p* < 0·001)
Potential contagion	2·00 (0·74)	1·92 (0·80)	1·84 (0·69)	0·09 (−0·03, 0·20) (*p* = 0·232)	0·16 (0·05, 0·27) (*p* = 0·002)	0·07 (−0·05, 0·20) (*p* = 0·420)	−0·18 (−0·26, −0·09) (*p* < 0·001)
ASABA	2·67 (0·52)	2·58 (0·57)	2·45 (0·51)	0·10 (0·03, 0·17) (*p* = 0·003)	0·23 (0·15, 0·30) (*p* < 0·001)	0·13 (0·05, 0·21) (*p* < 0·001)	−0·23 (−0·29, −0·17) (*p* < 0·001)
CUS	2·81 (0·70)	2·74 (0·73)	2·44 (0·51)	0·07 (−0·05, 0·18) (*p* = 0·456)	0·38 (0·26, 0·48) (*p* < 0·001)	0·30 (0·19, 0·41) (*p* < 0·001)	−0·22 (−0·31, −0·13) (*p* < 0·001)

^a^ Repeated measures ANOVA.

^b^ Paired-sample *t*-test using raw data for score changes (12-month minus baseline).

Table 7 shows the mean ASABA and CUS scores for girls and boys, and for age groups ≤17 and ≥18 years at baseline and 12-month follow-up at the intervention school. There was strong evidence of a difference between boys and girls in mean score at baseline for both the ASABA and the CUS, with higher scores among boys for both scales. At 12-month follow-up, however, there was no longer evidence of a difference between these groups. The score decrease for the ASABA between timepoints was 23.3% among girls and 31.2% among boys. For the CUS, the score decrease was 27.3% among girls and 24.3% among boys. There was also strong evidence of a difference between age groups in mean score at baseline for two ASABA subscales (“negative stereotypes” and “potential contagion”), with higher scores in the younger age group. There was no longer evidence of a difference at 12-month follow-up.

**Table 7.** Mean (SD) scores and score changes for boys and girls, and different age groups at the intervention school

Subscales of ASABA	Baseline		*p*-value ^a^	12 months		*p*-value ^a^	Change (95% CI)^b^	Change (95% CI)^b^	Baseline		*p*-value ^a^	12 months		*p*-value ^a^	Change (95% CI)^b^	Change (95% CI)^b^
	Gender		* *	Gender		* *			Age group		* *	Age group		* *		
	Girls	Boys		Girls	Boys	* *	Girls	Boys	≤17 y	≥18 y		≤17 y	≥18 y		≤17 y	≥18 y
Negative stereotypes	3·36 (0·82)	3·48(0·68)	0·060	2·22 (0·78)	2·21 (0·87)	0·940	−1·23 (−1·39, −1·09) (*p* < 0·001)	−1·36 (−1·51, −1·20) (*p* < 0·001)	3·47 (0·73)	3·22 (0·83)	<0·001	2·22 (0·83)	1·99 (0·59)	0·155	−1·29 (−1·40, −1·18) (*p*<0·001)	−1·46 (−1·79, −1·14) (*p*<0·001)
Discrimination and exclusion	1·67 (0·60)	1·92 (0·67)	<0·001	1·45 (0·43)	1·52 (0·48)	0·164	−0·26 (−0·36, −0·15) (*p*<0·001)	−0·46 (−0·58, −0·35) (*p*<0·001)	1·81 (0·64)	1·73 (0·68)	0·224	1·48 (0·44)	1·49 (0·55)	0·880	−0·37 (−0·46, −0·29) (*p*<0·001)	−0·27 (−0·58, 0·04) (*p* = 0.082)
Potential contagion	1·70 (0·70)	1·97 (0·83)	<0·001	1·46 (0·53)	1·52 (0·68)	0·360	−0·31 (−0·44, −0·19) (*p*<0·001)	−0·50 (−0·65, −0·35) (*p*<0·001)	1·89 (0·77)	1·64 (0·74)	0.001	1·47 (0·55)	1·33 (0·58)	0·210	−0·40 (−0·50, −0·30) (*p*<0·001)	−0·56 (−0·96, −0·15) (*p* = 0.009)
**Full scales**			** **				** **	** **	** **	** **	** **	** **	** **	** **	** **	** **
ASABA	2·43 (0·54)	2·63 (0·54)	<0·001	1·79 (0·49)	1·83 (0·58)	0·545	−0·70 (−0·80, −0·61) (*p*<0·001)	−0·87 (−0·97, −0·76) (*p*<0·001)	2·57 (0·52)	2·39 (0·60)	0.001	1·81 (0·52)	1·69 (0·48)	0·241	−0·79 (−0·86, −0·71) (*p*<0·001)	−0·85 (−1·13, −0·57) (*p*<0·001)
CUS	2·55 (0·83)	2·63 (0·54)	<0·001	1·91 (0·68)	1·99 (0·72)	0·271	−0·65 (−0·79, −0·52) (*p*<0·001)	−0·82 (−0·97, −0·67) (*p*<0·001)	2·67 (0·79)	2·69 (0·93)	0·760	1·95 (0·68)	1·82 (0·78)	0·351	−0·71 (−0·82, −0·61) (*p*<0·001)	−1·02 (−1·44, −0·61) (*p*<0·001)

^a^ Independent-sample t-test for changes between baseline and 12-months follow-up.

^b^ Paired-sample t-test for changes between baseline and 12-months follow-up.

## Discussion

The present quasi-experimental study showed that a school-based stigma-reduction programme promoting gender equality, equitable norms and value clarification could be effective in transforming attitudes and beliefs regarding abortion and contraceptive use among secondary-school students in Kisumu County, Kenya. Although the mean scores on both the ASABA and the CUS decreased at 1-month follow-up and 12-month follow-up compared with baseline at both schools, the percentage decrease was greater among students at the intervention school than among those at the control school. Our focus on adolescents emphasises the need to address the stigma of abortion and contraceptive use at an early age, as this may reduce the risk of negative health outcomes and social and economic consequences that can last a lifetime.

At the intervention school, stigma scores at baseline were higher for both the ASABA and the CUS among male students compared with female students. However, there was no evidence of difference between boys and girls at 12-month follow-up, meaning that there was a larger decrease in mean score among boys than among girls, particularly regarding abortion stigma. Although we did not pre-specify a threshold for effectiveness according to gender, we consider that the intervention could be effective in reducing stigmatising attitudes among both boys and girls.

The mean scores at baseline were high for the ASABA item related to perceiving abortion as a sin. In Kenya, most people are deeply religious, and abortion is often considered equivalent to murder, while contraceptive use among adolescents is considered wrong.^20,31,32^ Also, intercourse is seen as a practice for conceiving children rather than for pleasure,^35^ which could explain the relatively high mean score for the item on considering girls who use contraceptives to be promiscuous and a bad influence on other girls. Such attitudes could create a fundamental barrier to young women seeking contraceptive services.

CSE practices are often not aligned with the curriculum or with a liberal view on sexuality. In 2013, the Kenyan government committed to presenting a rights-based CSE programme, beginning in primary school.^16^ However, pregnancy-related topics are still neglected in CSE programmes; national guidelines are not implemented, and community members, including healthcare providers, are still unaware of their rights.^9,33^ The teaching messages are abstinence from sexual activity and that sex is immoral and dangerous for young people.^18,33,34^ Stigmatising attitudes discriminate against and silence young women regarding their sexual and reproductive health needs, and this leads to poor health outcomes.^10,33^

Unsafe abortion continues to be a major public health concern for many, and particularly young, women in Kenya and, therefore, stigma-reduction activities need to be addressed in the CSE curriculum. A recent review concluded that reduced access to reproductive health services could result in unsafe attempts to prevent conception or induce abortion, such as the use of battery acid and crushed bottles.^32^ Another survey showed that only 13–20% of students aged 15–17 years reported that they knew about modern contraceptive methods and their availability.^34^

In the formative work for this study, the research team learned that adolescent girls associated with abortion and contraceptive use were at risk of social judgement and discrimination by not only their peers but also their teachers^35^ and healthcare providers^20^ at youth-friendly clinics. However, during the process, we inferred that teachers’ and providers’ attitudes became more liberal. Therefore, teachers would benefit from receiving education and training on evidence-based adolescent sexual and reproductive health and how to implement the CSE programme effectively.

In the present study, there was a great deal of stigma among young people regarding reproductive choices such as abortion and contraceptive use. However, the implication of the current findings is that talking about abortion and contraception in a non-judgmental way can help to explore different values relating to adolescent pregnancy, contraceptive use and gender norms. These values must be explored if we are to find measures to prevent stigma and its toxic consequences. The school environment must provide opportunities to discuss sexual and reproductive health, in a safe space where young people can learn more about their own rights and responsibilities – not only in terms of medical information and legal facts, but also that sexuality is something they are permitted to talk about, which may help to reduce stigma around it. In particular, this can help them to access safe services in public facilities when they need them. Furthermore, the study demonstrates that relatively minor efforts could be effective. The results may be generalisable to Christian secondary schools in Kenya, as the study population reflects the broader populations of this age group in Kenya. However, further research is needed to assess the generalisability of the intervention to other geographical areas and cultures. This research project was planned and organised in collaboration with the local Ministry of Education, different stakeholders and adolescents. Therefore, the intervention may be further developed, adopted and implemented as part of broader efforts to improve CSE by the Ministry of Education in Kenya.

### Strengths and limitations

The inclusion of a control arm was a strength, even though the study was not set up for between-arm comparisons. Obtaining data at multiple timepoints at both schools, including pre-intervention, was also helpful in the assessment of potential intervention effects. However, conducting more than one pre-intervention measurement would have been preferable in terms of seeing whether the trends in the schools were in tandem before the programme was implemented. Although there were differences between the two study arms at baseline, the students are likely to have shared similar sociodemographic characteristics as both schools were located in poor peri-urban settlements, so the groups could be considered comparable. The same individuals with at least one follow-up were analysed, which gives strength to the conclusions regarding the potential effectiveness of the intervention. The tools used, ASABA and CUS, have been validated,^30^ and a self-administered procedure with a five-point Likert scale to measure attitudes and beliefs was used, which is an effective method for collecting information on a sensitive topic. Furthermore, this was a supervised classroom survey, and the research assistants could check that the respondents filled in their own questionnaires. The low internal drop-out rate for the data collection tools may reflect their validity. In terms of potential contamination between the study arms, the distance between the schools was approximately 5 km; owing to the restricted infrastructure and limited access to digital networks in the study setting, we consider the risk of contamination to be low.

However, the study did have some limitations. We were allowed to collect additional data only on age and gender because of the sensitive nature of the topic, and therefore we lacked information on other potentially relevant factors such as family situation and living conditions. In addition, the study design could be considered a limitation compared with a large-scale cluster-randomised trial set up to formally compare results between study arms; however, there were insufficient resources for the latter study design. Another potential weakness was the lack of a formal sample size/power calculation. In addition, participants who had left the school at 12 months were not included in the analyses, which could have affected the results. No attempts were made to follow-up those individuals owing to logistical constraints. The internal consistency of the ASABA subscales was fairly weak, and for the full CUS scale. This could be partly explained by the few items in each subscale. For the full scale, the consistency was acceptable.

Some focus group discussions took place with student peer counsellors (*n* = 21) and teachers (*n* = 20) at the intervention school before the baseline measure, which could account for some of the differences between the schools at baseline. In other words, the students at the intervention school could have been more aware of the study and felt more comfortable responding truthfully compared with those at the control school. Social desirability bias might also have been a factor for students who were more aware of the study objectives. To ensure meaningful comparisons, the results at the schools were not formally compared with each other. Instead, adolescents’ own test results were compared at different timepoints. Another consideration is the fact that the mean score also decreased over time at the control school for both the ASABA and the CUS, which may indicate low test–retest reliability for the scales. Using the same scale may have desensitised the participants’ attitudes towards these sensitive topics, a challenging fact considered during the validation process.^30^ It is also possible that the students’ opinions and views changed as they gained life experience on these topics or that other events happened outside the intervention that affected their responses. However, the score change was relatively small at the control school, whereas a large score change was observed at the intervention school. Therefore, the current study sheds light on adolescents’ need for a stigma-reduction programme within CSE as a key component of addressing stigma surrounding abortion and contraceptive use. In addition, stigma associated with abortion and contraceptive use are positively correlated, as also recently suggested by Blodgett et al^36^ – according to whom interventions addressing contraceptive use stigma may provide a socially acceptable approach for reducing abortion stigma by proxy. Our interpretation is that the findings emphasise a broader view on reproductive stigma and should, therefore, be addressed in a holistic manner.

## Conclusions

Stigma related to abortion and contraceptive use is often overlooked in CSE programmes. This study indicates that providing opportunities to discuss sexual and reproductive health, in a safe space, could be effective for teaching students about their own rights and their responsibilities. A non-judgmental approach enabled us to explore and address different values related to adolescent pregnancy, contraceptive use and gender norms. The material was not only about medical learning and legal facts, but also showed students that sexuality is something that they are allowed to talk about, which may help to reduce stigma around it. Larger-scale studies are needed to move forward in the development of pathways to address stigma associated with abortion and contraception, and these topics should become a priority for high-quality CSE policies, guidelines and programmes.

## Data sharing

The data that support the findings of this study are available on request from the corresponding author. The data are not publicly available because the data may contain information that could compromise the privacy of research participants.
